# Immersive virtual reality-based learning as a supplement for biomedical engineering labs: challenges faced and lessons learned

**DOI:** 10.3389/fmedt.2024.1301004

**Published:** 2024-03-19

**Authors:** Ishita Tandon, Vitali Maldonado, Megan Wilkerson, Amanda Walls, Raj R. Rao, Mostafa Elsaadany

**Affiliations:** Department of Biomedical Engineering, University of Arkansas, Fayetteville, AR, United States

**Keywords:** biomedical engineering education, augmented and virtual reality, undergraduate laboratory, optimized implementation, improving classroom teaching

## Abstract

**Introduction:**

Immersive virtual reality (VR) based laboratory demonstrations have been gaining traction in STEM education as they can provide virtual hands-on experience. VR can also facilitate experiential and visual learning and enhanced retention. However, several optimizations of the implementation, in-depth analyses of advantages and trade-offs of the technology, and assessment of receptivity of modern techniques in STEM education are required to ensure better utilization of VR-based labs.

**Methods:**

In this study, we developed VR-based demonstrations for a biomolecular engineering laboratory and assessed their effectiveness using surveys containing free responses and 5-point Likert scale-based questions. Insta360 Pro2 camera and Meta Quest 2 headsets were used in combination with an in-person lab. A cohort of 53 students watched the experimental demonstration on VR headsets in the lab after a brief lab overview in person and then performed the experiments in the lab.

**Results:**

Only 28.29% of students reported experiencing some form of discomfort after using the advanced VR equipment as opposed to 63.63% of students from the previous cohort. About 40% of the students reported that VR eliminated or reduced auditory and visual distractions from the environment, the length of the videos was appropriate, and they received enough information to understand the tasks.

**Discussion:**

The traditional lab method was found to be more suitable for explaining background information and lab concepts while the VR was found to be suitable for demonstrating lab procedures and tasks. Analyzing open-ended questions revealed several factors and recommendations to overcome the potential challenges and pitfalls of integrating VR with traditional modes of learning. This study provides key insights to help optimize the implementation of immersive VR to effectively supplement in-person learning experiences.

## 1 Introduction

STEM education has begun embracing immersive virtual reality (VR)-based laboratory displays. In addition to remote learning, VR may be a useful tool for outreach, waste reduction, safety improvement, piqued interest, and modernizing education. Virtual reality has a lot of potential to enhance current educational approaches (1, 2). Advanced utilization of the VR technique can provide virtual hands-on experience, an approach widely popular in the gaming industry, to facilitate experiential and visual learning and enhanced retention (3, 4). VR use in a classroom setting has proven to provide many advantages over generic lectures and 2D experiences. Despite its benefits, there are still many challenges that arise with the use of VR, including poor implementation, lack of time to master technology, and limited instructional content (4). However, it is crucial to carry out optimizations of VR implementation, in-depth analyses of benefits and trade-offs of the technology, and assessments of receptivity of contemporary methodologies in STEM education in order to ensure better utilization of VR-based education (1, 4, 5).

Several studies have tried to assess the effectiveness of VR in the realm of STEM education (6–10). For radiotherapy students, VR technology was used to simulate a radiotherapy treatment system. An increase in the student's comprehension of technical skills and their confidence in using them also improved (11). Another study that successfully improved the performance of students with low spatial abilities employed VR technology to teach chemical concepts to students (12). Then, a study used VR in business education and showed that students can gain a broad range of skills through this technology (13). A recent study by Singh et.al. employed VR as a tool to help teach communication skills in a clinical setting to biomedical engineering students. In this study, students reported feeling more immersed in “real-world” scenarios and were able to better understand their roles working on an interdisciplinary team after using VR (2). Not only can VR improve learning through emotional experiences, but there have also been studies that report the efficacy of VR in teaching technical skills such as data visualization, engineering design, and even surgery (4).

In a previously published study (14), we created VR-based demonstrations for a biomedical engineering lab and evaluated their efficacy using surveys with open-ended questions and questions based on a 5-point Likert scale. In a cohort of 56 students, more than 70% said that VR films gave them more pacing and task understanding flexibility, however, 65% of the students said they felt some discomfort. Overall, following VR-based demos, students greatly improved on lab quizzes. Using Insta360 EVO VR camera in 180° 3D mode, 20–50-min-long laboratories with an overview and experiment were recorded and visualized by students via the Google Cardboard headsets (14).

The goal of the current study was to use VR as an additional method of lab instruction by overcoming the constraints of video length and equipment quality. Modern VR tools, such as the Insta360 Pro2 camera and Meta Quest 2 headsets, were combined with the in-person lab. The study's objectives were to evaluate how the students perceived and used VR in terms of its potential for usage in the future, engagement, content, and functionality. This study's insights aid in maximizing the use of immersive VR to successfully enhance in-person learning experiences and avoid the possible problems of combining VR with conventional modes of learning.

## 2 Materials and methods

### 2.1 Experimental design and lab overview

With the goal of assessing the applicability of VR technology to supplement in-person labs, 53 students in a junior-level biomolecular engineering lab in the Department of Biomedical Engineering were exposed to VR videos. Before the students arrived at the lab, they had to complete a pre-lab quiz for each lab. The students were given a brief rundown of the lab's topics (Table 1) and background material as well as a thorough explanation of how to conduct the experiments. To enable the groups of students to collaborate on the experiments, the class was divided into groups of 2–4 students. Students were required to complete a post-lab assessment a few days after the conclusion of each lab session. The students were also required to present a lab report outlining the background, procedures, findings, analysis, and discussion after every two laboratories which were graded according to the rubric outlined in Appendix A (Supplementary Appendix A, Table A.1).

**Table 1:** Topics taught in each lab.

Lab	Lab topic	Description
1	Bacterial growth	Spectrophotometer is used to determine the turbidity and therefore concentration of batch-cultured E. coli at specific time points followed by formulation and interpretation of bacterial growth curve.
2	Bacterial transformation	E. coli are made competent using CaCl_2_ and heat shock technique and transformed using a recombinant plasmid. Cells are then cultured on agar plates to verify transformation using antibiotic resistance imparted by the plasmid.
3	DNA isolation and Purification	Transformed E. coli are used to isolate plasmid using an alkaline lysis assay and the plasmid concentration and purity is determined by performing gel electrophoresis.
4	Restriction Digestion	The isolated plasmid is subjected to restriction digestion using 2 separate restriction endonucleases or their combination. Gel electrophoresis is performed to assess the properties of digested and undigested plasmids.
5	Protein Quantification	A Bicinchoninic acid (BCA) assay and UV absorbance method is employed to determine the concentration of unknown proteins.
6	Protein purification	Affinity chromatography is used to purify and elute proteins isolated from bacterial cell culture.
7	Protein Concentration	Centrifugal filtration method is used to concentrate the isolated proteins from lab 6 which are then quantified using BCA assay.
8	SDS-PAGE	The concentrated samples from Lab 7 are separated using Sodium dodecyl sulfate-polyacrylamide gel electrophoresis (SDS-PAGE) and assessed.

The first four in-person labs, which were referred to as pre-VR labs, did not use VR videos. These pre-VR labs entailed an overview of the lab along with the lab procedures delivered to the students via traditional PowerPoint slides on a projector screen at the beginning of each lab. The VR-based videos were included in laboratories five and later, which were referred to as post-VR labs. Students were instructed to utilize the VR headsets to watch the movies at the start of each lab and apply the information offered there to carry out the lab procedures. The video's step-by-step instructions for the experiment that will be carried out in that lab were developed by the lab teachers. The lab instructors were on hand the entire time to help the students watch the videos, use the VR equipment, and carry out the lab experiments.

The outcomes of this study were measured using two surveys. Pre-VR survey was presented after lab 4 concluded and post-VR survey was presented after the conclusion of lab 8. Each participant in this study gave their agreement to take part. This research was approved by the University Institutional Review Board (IRB protocol #: 2012306663).

### 2.2 Virtual reality technology and video creation

**Figure 1:**
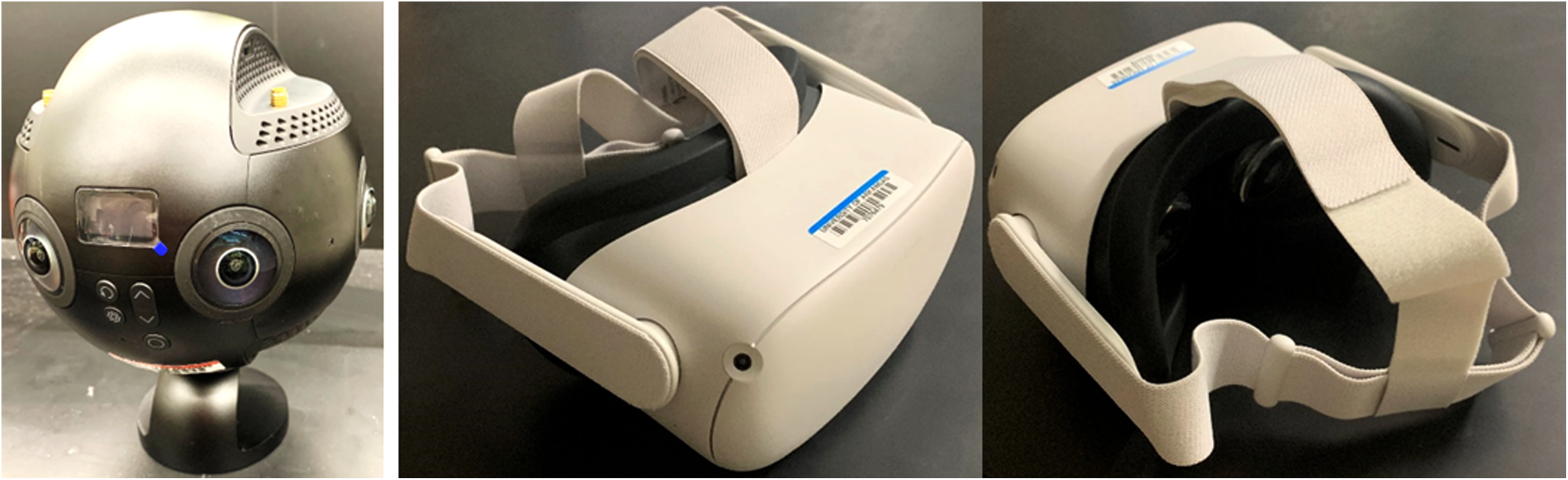
Insta360 Pro II camera used to film the VR videos in a 360° format. Front and back profiles of the Pro Quest 2 VR headset used to view the content in a VR format.

Before filming the videos, a media production manager within the university trained the four lab instructors (2 graduate and 2 undergraduate students) to film and edit the videos. The first training entailed the know-how of using the camera and increasing the video quality by optimizing the filming distance, transitions, camera movement, and video editing. The camera used to film the VR videos was the Insta360 Pro II (Figure 1). The headsets used for this study were the Oculus Quest 2 VR headsets from Meta (Figure 1). Adobe Premiere Pro was used for editing VR videos and is available for free for the teaching assistants from the university. One Insta360 Pro II Spherical VR 360 8 k Camera was purchased and its total cost was $4,599.00. One Oculus Quest 2—Advanced All-In-One Virtual Reality Headset amounted to $299.00. To accommodate the maximum number of students enrolled in a single lab period 20 headsets were purchased. Their total cost amounted to $5,980.00.

For filming, the camera was placed in a static position for each video filming to minimize the discomfort of the user. The camera was placed in a position so that the video could capture the teaching assistant and experiment to include all the details. Once the videos were filmed and edited, the 3–20-min VR videos (Table 2) were uploaded into each headset individually for students to watch during their respective lab time. All the students were able to watch the video at the same time on a personal headset.

**Table 2:** Length of the videos in minutes by cohort.

Lab video duration		
Lab number	Cohort 1	Cohort 2
1	10:45 min	NA
2	37:56 min	NA
3	37:33 min	NA
4	19:51 min	NA
5	27:50 min	8:01 min
6	19:46 min	17:05 min
7	12:55 min	3:15 min
8	13:57 min	17:24 min
9	22:11 min	NA

To compare how the quality of the VR equipment alters the student experience of the VR-based labs, students from two different semesters of the same lab were considered as cohort 1 (*n* = 56) (14) and cohort 2 (*n* = 53). For cohort 1, the Insta360 EVO VR camera in 180° 3D mode was utilized to record 20–50 min-long labs (Table 2) incorporating a brief overview and experiment and visualized via Google Cardboard headsets (14). Insta360 EVO camera costs about $500. The Google Cardboard (VR Headsets 3D Box Virtual Reality Glasses) cost $9.99 per headset amounting to a total cost of $639.36 for a total of 64 headsets that were purchased (14).

### 2.3 Survey creation and distribution

Two surveys containing open-response questions (mentioned in this manuscript within “”) and 5-point scale Likert questions with the options of “Strongly Disagree”, “Disagree”, “Neutral”, “Agree”, and “Strongly Agree” were designed. Likert questions were converted into 1–5 respectively for data analysis purposes. The first survey (Pre-VR) contained additional true/false questions. After the students finished the first four experiments, they were given the first survey, which was designed to gauge their expectations for using VR technology (Supplementary Appendix A, Table A.2). The second survey (Supplementary Appendix A, Table A.3), which was given out at the conclusion of the semester, was designed to gauge how the students felt about using virtual reality (VR) technology as extra course material after viewing the videos.

### 2.4 Data analysis

Percentage responses were calculated for all survey questions and data was represented as histograms. Nonparametric tests were used for all comparisons. Direct comparison between pre- vs. post-surveys was conducted using paired samples Wilcoxon signed-rank test. Kruskal–Wallis' test with Dunn's multiple comparisons was used for the grades obtained in lab reports. Friedman's test was used to assess the pre- and post-lab quiz scores for cohort 2. Comparisons between cohort 1 and cohort 2 post-VR surveys and post-lab quiz scores were conducted using the Mann–Whitney test. Power analysis was performed and a *p*-value of <0.05 was considered significant. Lab report and quiz scores were represented as median with minimum and maximum data. Quantitative data was exported to GraphPad Prism for statistical analysis and graphical representation. Comparison between the comments in the open response questions as well as the true/false questions were considered in the data analysis to determine the degree of effectiveness of VR technology as supplemental teaching tool to in-person labs. The responses to the open-ended questions received have not been altered while being reported in the paper and may include spelling mistakes, grammatical errors, and slang.

## 3 Results

### 3.1 Students thought that VR may make the course more interesting but only a few felt comfortable/familiar with VR-based labs

**Figure 2:**
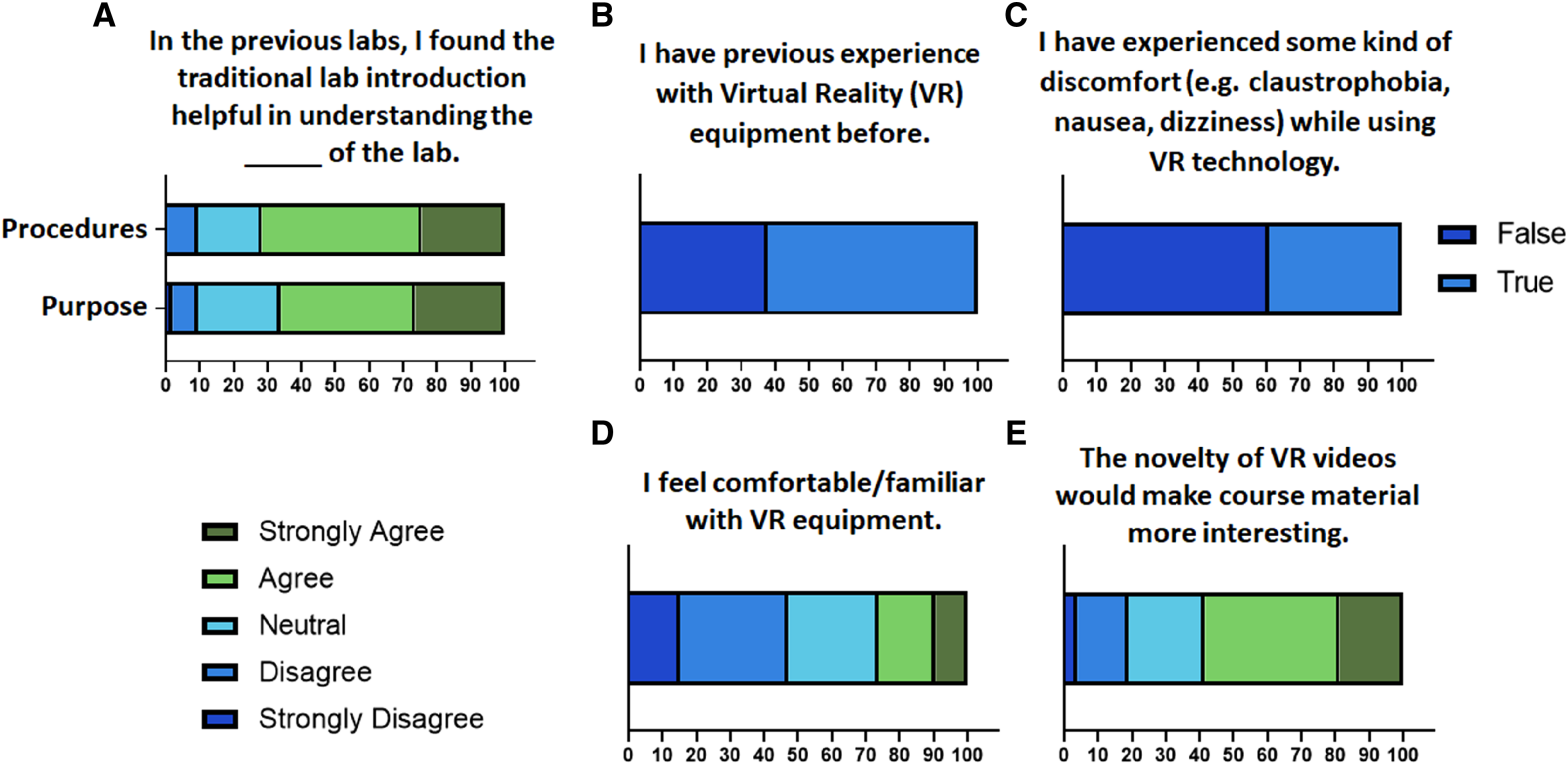
Assessment of student perceptions about the utility of traditional methods and their comfort and familiarity with VR technology. Contingency plots and pie charts representing the percentage of student responses about their (**A**) perception of the utility of traditional labs, (**B**) prior experience with VR equipment, (**C**) experience of discomfort while using VR previously, (**D**) comfort/familiarity with VR equipment (**E**) expectation from the novelty of VR to make course material interesting.

After the first four labs were delivered via traditional methods, a Pre-VR Survey was analyzed to assess the perception of students about their familiarity and expectations pertaining to VR and traditional methods. Most students agreed or strongly agreed that the traditional lab introduction was helpful in understanding the purpose and procedures of the lab (Figure 2A). Out of the 53 students, 62.26% of students admitted to having some form of previous experience with VR equipment (Figure 2B); however, 39.39% of those students said to have experienced some kind of discomfort (e.g., claustrophobia, nausea, dizziness) while using VR technology previously (Figure 2C). Overall, only 9.43% of the students strongly agreed and 16.98% students agreed that they felt comfortable/familiar with VR equipment before experiencing VR in this lab (Figure 2D). These results suggested low enthusiasm and apprehension among the students before taking VR-based labs. However, 58.48% of students agreed or strongly agreed that the novelty of VR videos would make the course material more interesting (Figure 2E).

Based on the open response question “What are your expectations for the VR videos?”, only 25 out of 53 students seemed to have a positive/hopeful notion before experiencing the VR-based labs. These responses included “They will be pretty cool”, “I expect the VR videos will be helpful in teaching proper lab techniques for the experiemnts. Additionally, I think that the VR videos will give us a good idea of what to expect before starting the actual experiment.”, “Excitement!”, “I think it will be a new fun way to learn”, “realistic, practical”, and “That they aid in our retention and give us a more immersive view”. These positive comments included 3 specific mentions of the expectation for VR to be interactive.

Low enthusiasm and apprehension among the students were further corroborated by the open-response questions summarized in Supplementary Appendix B.1. A few students weighed in on where they see the utility of VR videos as opposed to the traditional lab methods via comments summarized in Supplementary Appendix B.2. The students also revealed the specific features of the traditional labs they liked via comments summarized in Supplementary Appendix B.3. Overall, the ability to interact with the lab instructor, have them answer questions and be able to refer to the introductory material seemed to be the most desirable attributes of the traditional lab format.

### 3.2 The use of VR did not positively impact student engagement, material understanding and retention, and transferability of learned skills as compared to traditional labs

To assess the student perception vs. their experience of VR-based labs, pre- and post-lab surveys were compared directly. Based on the student responses to Likert-type questions, the effect on student engagement, material understanding and retention, and potential for use of VR-based learning in the future were assessed. Analysis of discomfort experienced by students while using VR was also performed and compared to the pre-VR survey. For that, 5-point Likert-type responses in the post-VR survey were converted to binary responses for direct comparison with true/false responses from the pre-VR survey. Strongly agree and agree responses were considered as true, strongly disagree and disagree were considered as false while neutral responses were not considered for this analysis.

**Figure 3:**
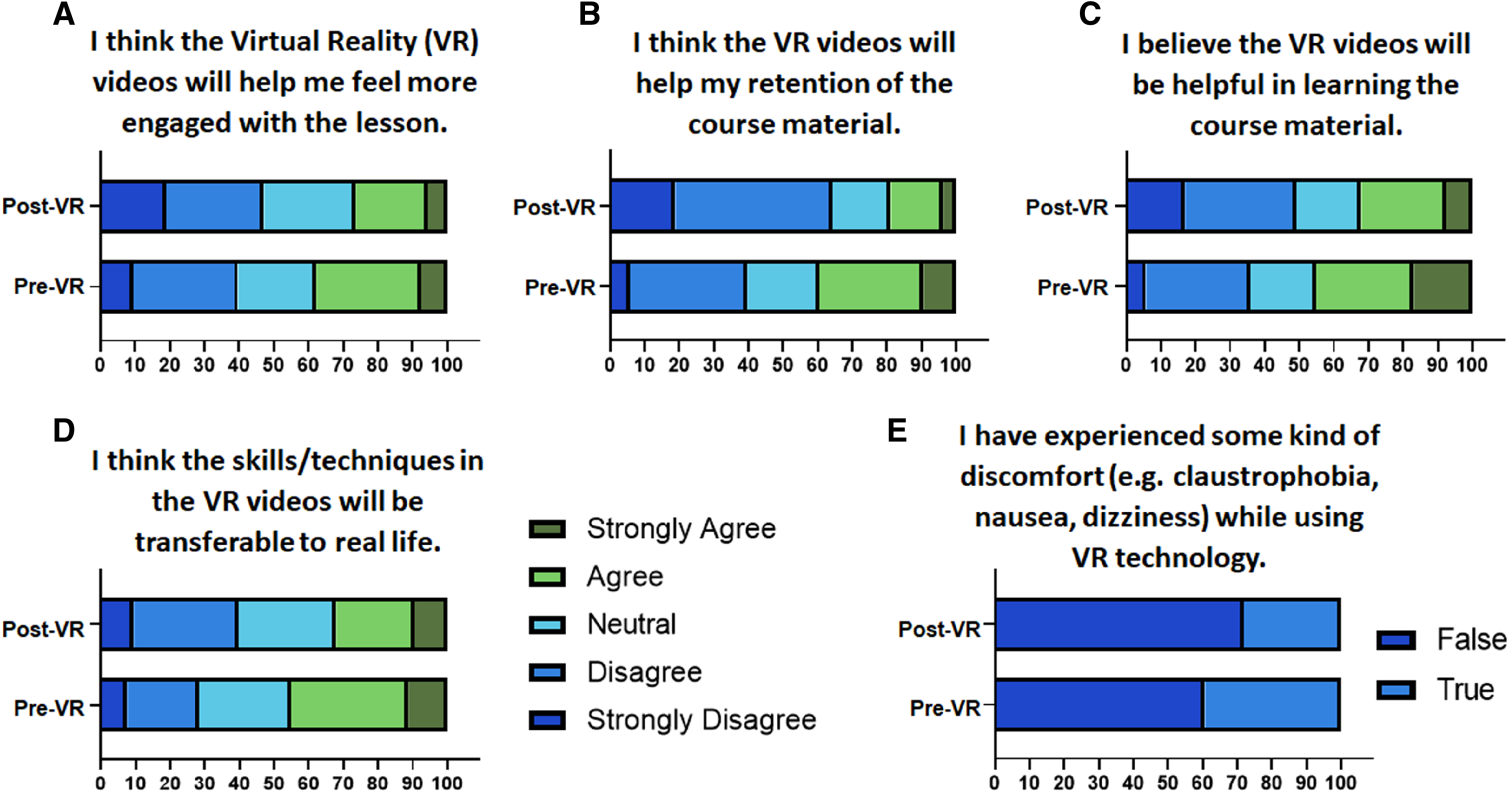
Direct comparison of student perception vs. their experience of VR-based labs. Contingency plots representing the percentage of students who responded to questions assessing the effect of VR on (**A**) student engagement, (**B**) material retention, (**C**) material understanding, (**D**) transferability of learned skills, and (**E**) experienced discomfort.

The percentage of students who agreed or strongly agreed that the use of VR helped them feel more engaged decreased significantly (*p* = 0.03) (Figure 3A). The percentage of students who agreed or strongly agreed that the use of VR helped them retain the course material also decreased while the percentage of students who disagreed or strongly disagreed, significantly increased (*p* < 0.0001) (Figure 3B). The percentage of students who agreed or strongly agreed that the use of VR helped them understand the course material decreased (*p* = 0.0049) (Figure 3C). Additionally, fewer students felt confident in applying the skills/techniques from the videos in the lab as opposed to before taking the VR labs (Figure 3D). While 39.39% out of 33 students with prior VR experience admitted to having discomfort while using VR in the pre-VR survey, only 28.29% of the 53 students said so after these VR labs (Figure 3E). This suggested that while the use of advanced VR equipment in this lab helped in reducing discomfort, other factors like content and implementation of VR in this lab may have influenced student engagement, material understanding and retention, and potential for use of VR.

### 3.3 Post survey revealed effective and unfavorable aspects of VR-based labs

**Figure 4:**
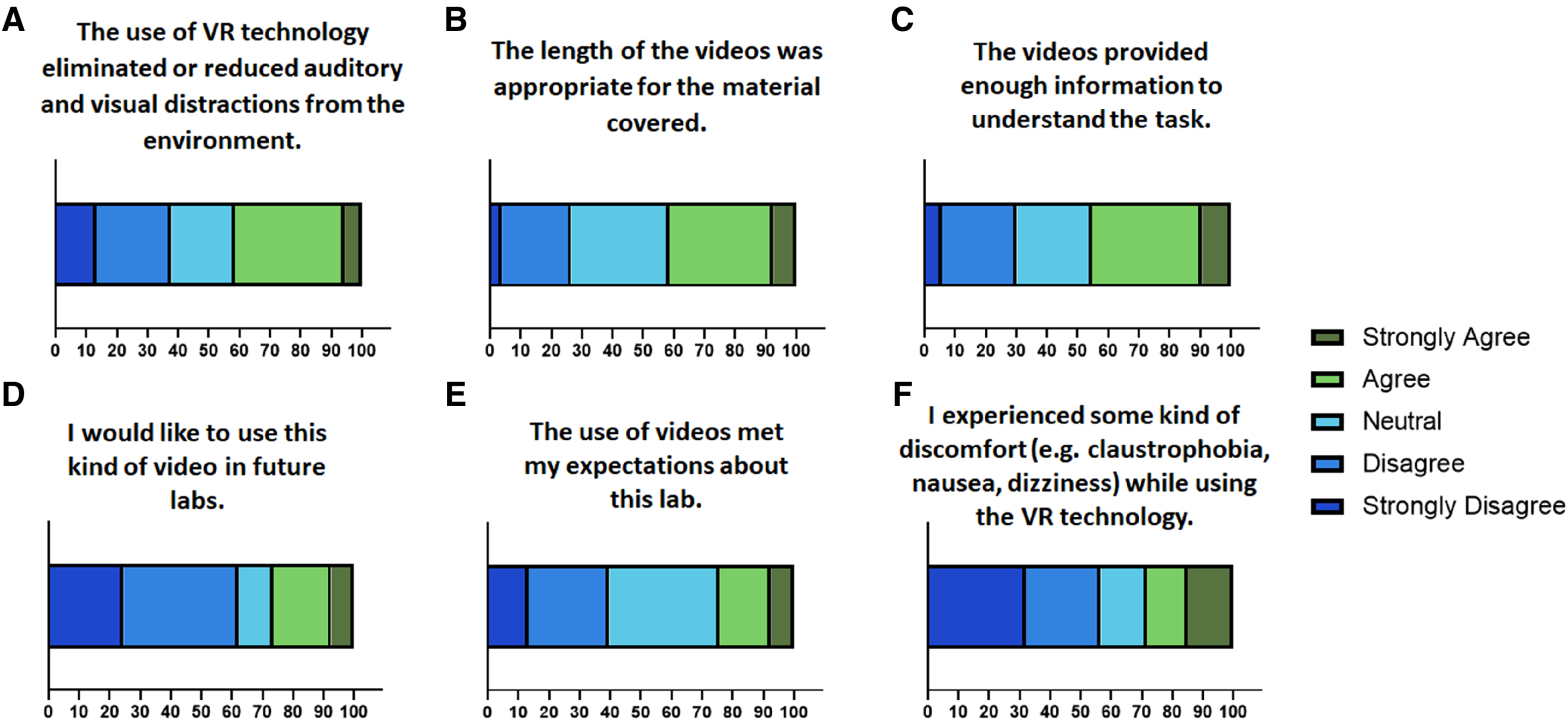
Contingency plots representing the percentage of students who responded to questions pertaining to the (**A**) engagement, (**B**) length of videos, (**C**) content of videos, (**D**) potential for future use, (**E**) meeting of expectations about the lab, and (**F**) discomfort experienced in the post-lab surveys.

To further assess engagement, potential for future use, and functionality of the VR technology used in the VR-based labs, Likert questions in the post-VR survey were assessed. In the post-VR survey, 41.51% of students agreed or strongly agreed and 37.74% of students disagreed or strongly disagreed that VR technology eliminated or reduced auditory and visual distractions from the environment (Figure 4A). Length of the videos (Table 2) was found to be appropriate by 41.51% of the students only (Figure 4B). When asked if the videos provided enough information to understand the tasks, 45.28% of students agreed or strongly agreed and 24.53% of students remained neutral (Figure 4C). Only 26.41% of students agreed or strongly agreed that they would like to use this kind of video in future labs (Figure 4D). However, when they were asked if the use of videos met their expectations about this lab, 24.52% of students agreed or strongly agreed, 35.85% remained neutral, 16.98% disagreed and 7.55% strongly disagreed (Figure 4E). After taking the VR-based labs, only 28.29% of students admitted to having experienced some kind of discomfort (e.g., claustrophobia, nausea, dizziness) and only 15.09% of students remained neutral (Figure 4F). This further suggests the use of advanced VR equipment enhanced the comfort level associated with the use of VR.

In response to the question “Did you experience any problems using/viewing the videos for the lab? If so, which ones?” 27 students reported experiencing no problems using the VR. Out of the other 26 students, only 5 students experienced discomfort like “made me dizzy”, “I just get motion sick in VR, and the camera was really far away”, “The video was very blurry which caused nausea”, “Not really, through the 3rd video gave me a headache.”, “The only problem I had is that I use glasses so it's a little bit uncomfortable.” The rest of the students reported experiencing technical issues associated with accessing and visualizing the VR videos correctly, like “I could not experience the videos as VR, I just could see a flat screen in the middle of an island. It was really hard to see the small details”, “Sometimes when I tried playing the video, it showed the two views on a screen instead of it actually being 3D and having one view for each eye.”, “The only problem that I experienced was that it did not really look it was a 3D environment. Sometimes I would see the video far away and I could not fix it to where the video would be an appropriate distance for me to view it.”, “Initially, it was hard to find the proper VR video in the files. Also, the VR was somewhat blurry and hard to adjust.”, and “My videos often just showed up as two separate videos. It didn't seem to use virtual reality. The activities that the TAs were demonstrating were very small”.

In response to the question “What aspects of the VR lessons were helpful and/or effective?”, 39 out of 53 students implied that being able to visualize the step-by-step details of the procedure and hear the walk-through from the instructor, was helpful and/or effective. These responses are worded like “It was helpful to see which chemicals were used and how they were integrated into the lab.”, “Being able to see steps of the lab being performed ahead of time was helpful.”, “Being able to see all of the steps of the experiment”, “They were concise and informational. So much so that not a lot of questions needed to be asked to understand the task.”, “Gave an up-close perspective of the lab procedures.”, “Being able to hear the TA up close.”, and “Seeing how the equipment was used beforehand was very helpful, since I had never worked with some of this labware before.”. Some other insights into the helpfulness of VR implementation were “that it allowed you to watch the process as a single viewer, not crowding around with the rest of the class”, “I liked the replay ability”, “Eliminating distractions”, and “It was easy to use”.

### 3.4 The content of the videos and the quality of VR equipment contributed to the student experience with VR

Students in the current study comprised cohort 2 for whom Insta360 Pro2 camera and Meta Quest 2 headsets, were used in combination with an in-person lab. Likert questions from the post-VR surveys were used to compare the content, potential for future use, engagement, and functionality of the VR equipment used for cohorts 1 and 2.

**Figure 5:**
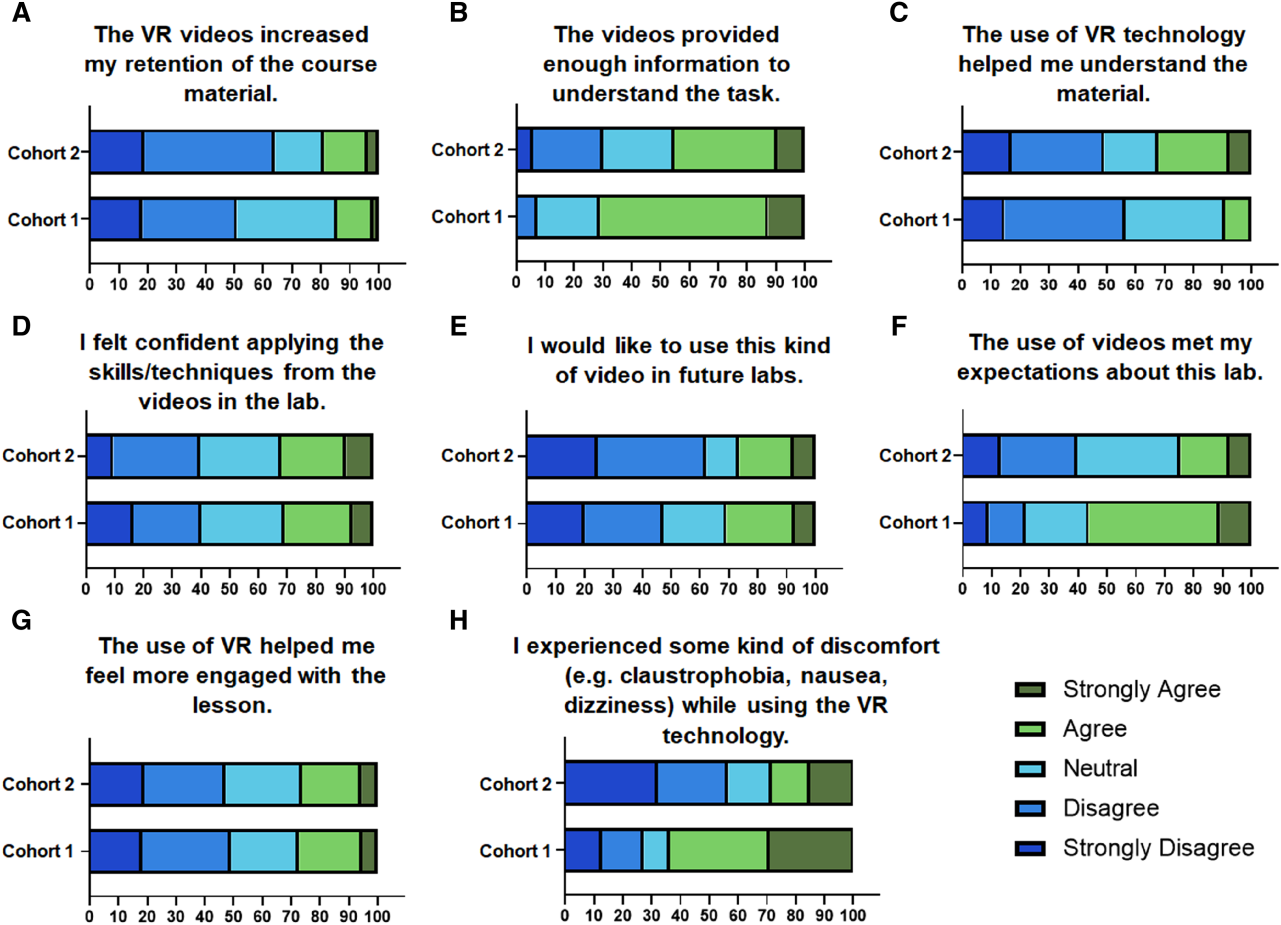
Comparison of student experience of VR labs-based labs from two different cohorts. Contingency plots representing the percentage of students who responded to questions assessing the effect of VR on (**A**) retention of material, (**B**) content of the videos, (**C**) material understanding, (**D**) applicability of skills, (**E**) potential for future use, (**F**) meeting of expectations, (**G**) student engagement, and (**H**) discomfort experienced.

The students were asked if the use of VR helped increase the retention of the course material. Interestingly, the number of students who agreed or strongly agreed increased from and the number of students who disagreed or strongly disagreed also increased in cohort 2 (Figure 5A). In response to the question that the videos provided enough information to understand the task, compared to cohort 1 (70.90%) a fewer number of students agreed or strongly agreed (45.28%) in cohort 2 while a greater number of students disagreed or strongly disagreed (Figure 5B). Additionally, a greater number of students in cohort 2 (32.08%) agreed or strongly agreed that the use of VR technology helped them understand the material as compared to 9.09% in cohort 1 (Figure 5C). This suggests that significantly more students understood the task (*p* = 0.0040) in cohort 1 based on the content of the videos, while the use of VR did not have a significant impact on the understanding of the material by the students. These responses may suggest that not only did the quality of content created by the lab instructors vary between cohorts 1 and 2 but also it may have also negatively influenced the experience of the cohort 2 students. However, the use of advanced VR equipment positively impacted the delivery of the content to the students. Consequently, the percentage of students who felt confident applying the skills/techniques from the videos in the lab did not alter between the two cohorts (Figure 5D). This further suggests that while better VR equipment may add value in terms of content and potential for future use, the quality of the content delivered by the lab instructors is a significant factor influencing the understanding and retention of the material. In cohort 2, more students disagreed or strongly disagreed with using these kinds of videos in future labs (Figure 5E). Significantly fewer students agreed or strongly agreed that the use of videos met their expectations about this lab (*p* = 0.0047) (Figure 5F). Interestingly, the difference in the equipment did not alter the students' response to the question that “if the use of VR helped them feel more engaged with the lesson” (Figure 5G). However, as expected, the advanced VR equipment was found to significantly reduce the discomfort associated with the use of VR among cohort 2 students (28.29%) as compared to cohort 1 students (63.63%) (*p* = 0.0007) (Figure 5H).

The differences in the video content, explanation of concepts, and teaching styles were further revealed by the post-VR survey open-response questions and student comments summarized in Supplementary Appendix B.4. Cohort 1 and Cohort 2 responses varied immensely for the post-VR survey “What aspects of the VR lessons were helpful and/or effective?”, “What aspects of the VR lessons were not helpful or effective?”, and both pre- and post-VR survey question “Suggestions or comments?”.

### 3.5 The use of VR aided in the understanding of lab procedures and tasks but not the lab concepts and background

Students were given a pre- and post-lab quiz before and after every lab to assess their knowledge of lab concepts and background information. They were also asked to write lab reports after every 2 labs which aided in the assessment of the knowledge of lab procedures and tasks. The effect of VR-based instructions and VR equipment on student learning was also assessed by comparing the pre- and post-lab quiz scores and the grades of the lab reports.

**Figure 6:**
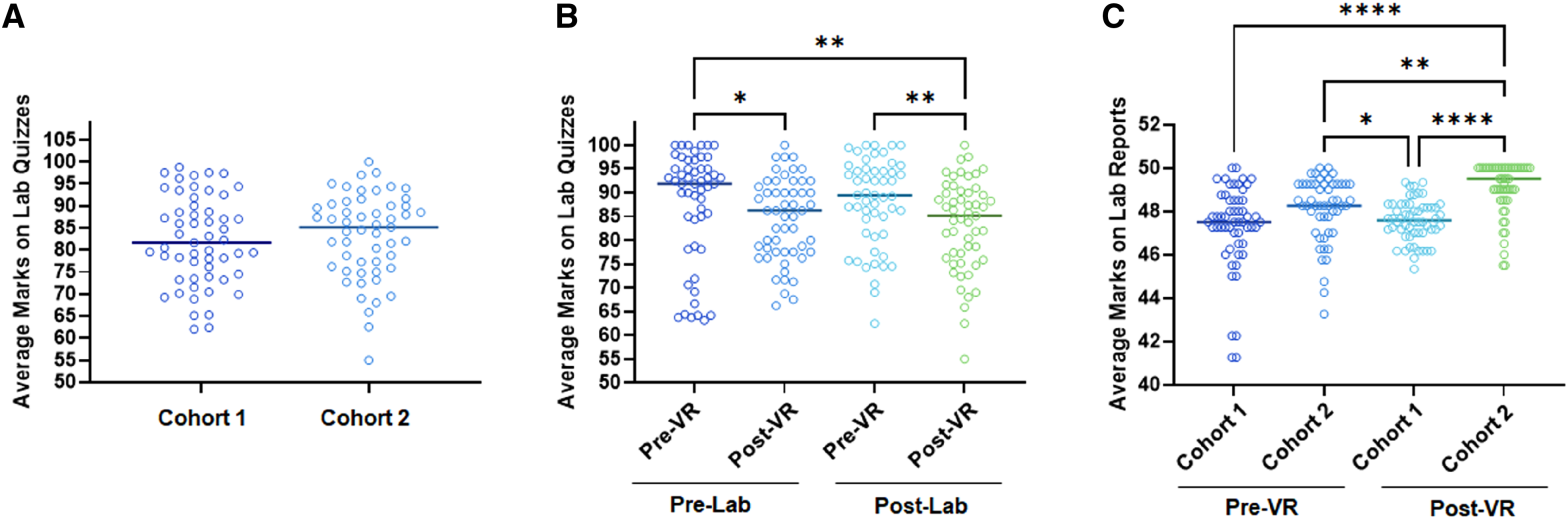
Assessment of the utility of VR in biomedical labs based on the lab quiz and lab report scores. (**A**) Average grades of students in post-lab quizzes in cohort 1 and cohort 2. (**B**) Average grades of cohort 2 students in pre- and post-lab quizzes from the pre- and post-VR labs. (**C**) Average grades obtained by cohort 1 and cohort 2 students in pre- and post-VR lab reports.

Between cohorts 1 (14) and 2, post-lab quiz scores for the VR-based labs were not significantly different suggesting that the equipment quality may not have impacted the material reception and retention of the lab concepts and background information (Figure 6A). Within cohort 2, students' scores on the pre-lab quizzes were not significantly different from the post-lab quizzes. Within the pre-lab quizzes, the student scores were not significantly different between the quizzes administered in post-VR labs (Labs 5–8) and pre-VR labs (Labs 1–4) (Figure 6B). This is expected as the students watched the VR videos in class after submitting the pre-lab quizzes. Interestingly, within the post-lab quizzes the student scores were significantly lower in the post-VR labs as compared to the pre-VR labs (Figure 6B). This may suggest that the use of VR in labs distracted the students from understanding and retaining lab concepts and background information.

Students in cohort 2 scored significantly higher in lab reports for the VR-based labs as compared to non-VR labs and compared to cohort 1 VR-based labs (Figure 6C). This may suggest that VR videos may have provided a better understanding of the lab procedures and tasks leading to a better-written lab report. It is although important to note that VR videos were given from lab 5 onwards, signifying that the students had received lab report scores and feedback on labs 1–4. That feedback may also be an additional factor further enhancing the quality of lab reports from lab 5. However, better VR equipment may have further enhanced the understanding and retention of the lab tasks which aided in better-written lab reports.

## 4 Discussion

After demonstrating great promise for future applicability and acceptance in the healthcare industry and education (10), VR is currently being assessed as a mode of education in the biomedical engineering field (2, 4, 14). This study aimed to assess the effect of VR as an in-lab supplement to the traditional mode of instruction for a biomedical engineering lab. This study unraveled several important facets of implementing VR as a complement to the traditional mode of instruction in a biomedical engineering-based lab, like understanding the 1. perceptions and experience of students before and after using VR based lab instructions, 2. value that VR based instruction may add to the learning experience, 3. effect of using an affordable but low-quality equipment vs. a high-quality equipment for VR recording and visualizations, and 4. other factors that may influence the VR based learning experience of the students.

The quality of the content of the videos was assessed by the question that “if the videos provided enough information to understand the task” while the ability of the VR to able to successfully deliver the video content was assessed via the question that “if the use of VR technology helped them understand the material”. The responses suggested that not only did the quality of content created by the lab instructors vary between cohorts 1 and 2 but also it may also have negatively influenced the experience of the cohort 2 students. However, the use of advanced VR equipment positively impacted the delivery of the content to the students. Additionally, Cohort 2 students scored lower in the post-lab quizzes in the pre-VR labs as compared to the post-VR labs. This is in stark contrast to the cohort 1 students who scored significantly better in the VR labs as opposed to no VR labs (14). These opposite outcomes between cohorts 1 and 2 may be attributable to the difference in the teaching styles of cohort 1 and 2 lab instructors, content created by the different lab instructors for lab concepts and background information, the structure of the labs with cohort 2 being in person and cohort 1 being remote, and how the VR-videos were incorporated in the labs (9). This further suggests that while better VR equipment may add value in terms of content and potential for future use, the quality of the content delivered by the lab instructors is a significant factor influencing the understanding and retention of the material. While there are cost based trade-offs with using a higher quality headset for each student (15), it is important to note that these costs would eventually go down with growing popularity and usage of VR (9). That cost is also offset by the supplies and compensation for lab instructor's hours. These labs require a lab instructor to show the experimental procedure to each student group multiple times during a single lab period, using the material and extending the time. Additionally, each lab instructor runs the labs prior to the lab period to ensure that no further troubleshooting is required. Having VR videos and experimental procedures captured on the video with all the specific details would eliminate the need for extra prior runs and repetitions in the lab period. Not only does that save lab instructor's time but also resources, resulting in overall cost. Furthermore, having an instructor record these videos for all students can ensure inter cohort and intra cohort variability in teaching styles. It can also enhance the quality of the content taught overall by utilizing the instructor's skills and focusing on the content quality.

Higher lab report scores and lower post-lab quiz scores suggest that VR videos may be more useful in demonstrating the lab procedures and details of the experiments while a more traditional approach of PowerPoint-based presentations may be more conducive to explaining lab concepts and background information. Such a conclusion is also supported by the open-ended responses like “I would rather have the TAs explain the lab to us in person.”, “I think demonstrating the techniques in VR is helpful but I think explanation of the concepts and ideas out of VR would be beneficial.”, “The TAs did a great job at explaining the labs via PowerPoints earlier in the semester. That method of explaining the lab helped me learn better”, “I prefer the slideshows the TAs show before the lab. I also think it is very helpful for them to walk us through as we go.” and “I think information being provided during the video lessons were not as helpful as when the TA directly talks about it and uses slides”.

Perception about the technology and prior experience with technology have also been reported as key factors in facilitating the integration of technology into instruction (16, 17). In this study, 28 out of 53 students did not have a positive/hopeful attitude towards incorporating VR into the labs as revealed by the pre-lab survey. Consequently, when a direct comparison was made between the students' perception and their experience, fewer students felt that VR positively impacted engagement, retention, and understanding of the material or met their expectations and was worth it for future use. This can be explained by the observations that 1. negative preconceived notions may contribute to the negative experience (18, 19) and 2. Negative reviews or notions about a product or its attributes tend to hold a stronger influence on the usability and satisfaction associated with a product as compared to positive reviews or notions about the same product or its attributes (20). Therefore, reduced discomfort offered by the better equipment, prior training of the lab instructors on VR equipment, and lessons learned from the previous study (14) were likely overshadowed by the negative preconceived notions and student expectations. These notions may have resulted from the prior experience of the students with VR, prior negative experience with VR, and/or casual discussions between cohort 2 students and cohort 1 students or lab instructors as evidenced by pre lab surveys and comments like “I might be wrong but i understood that instead of the introduction given by the TA, will have videos. I have heard that is even more confusing. So it would be great if we could have both”. Further, open questions and comments from students also pointed towards the apprehension of students about the advancement of VR technology and its utility in the Biomedical Engineering education field itself. To that extent student commented “I believe that there should be pre-lab videos where we can see the experiment (optional) before lab. However, no VR should be used. It's a cool concept, but for a lab like this it doesn't really serve a purpose.”, “VR is not at the level that it will be more useful than traditional instruction”, “I do not believe the use of VR is necessary when we have TA's that can help with the lab in person.”, “If the instructors are not practiced at it, quality will suffer.”, “I think it just makes sense to stick w the traditional way of doing it”.

Indeed, other studies when assessing VR-based instruction reported similar concerns like discomfort, technical issues, and distractions due to novelty (8) and discussed various challenges faced during implementing VR (7, 21–25). Such challenges and other contributors have impeded the process of incorporating technological advancements into the traditional education paradigm (26–28). Several reasons for the low acceptance and slow integration of new technologies, specifically VR, in education have been previously discussed (29–31). The insights from this study also revealed several factors that may help in the more efficient incorporation of VR-based learning into the traditional way of delivering STEM labs. Based on the quantitative data and student responses (mentioned in “”) to open ended questions, we summarize several recommendations and lessons learned from this study:

1. To overcome the technical issues faced by the students while using VR headsets, proper training, and a user manual could be provided to the students. This may reduce the interference of technical issues with the engagement and retention of the material. It may also increase familiarity and decrease distractions due to the technology itself. This insight was backed by the student comments like “The novelty of VR can be somewhat distracting and it can be somewhat difficult to find an open-enough area to watch the videos.”, “at first it was hard to locate the videos”, and “My videos often just showed up as two separate videos. It didn't seem to use virtual reality. The activities that the TAs were demonstrating were very small”.
2. The use of voice-overs, higher quality mics for recording the labs, or adjusting volumes appropriately during editing is an important factor to remember as the equipment in the lab like the biosafety cabinet, spectrophotometer, etc. can be loud. Some students commented “Have voice-over instead of room audio for video, better headset speakers, or separate audio devices (earbuds?) along with the video.”, “sometimes hard to hear the video”, “The audio was hard to hear so it kind of defeats the purpose” and “It was often difficult to hear, even with max volume”.
3. Zooming in on specific equipment, reagents, and materials used and clarifying those specifics may further enhance the utility of VR. Specific equipment, reagents, and materials can also be further clarified by adding captions, side notes, and disclaimers in the video during the editing process. Comments like “Subtitles may be helpful or like a zoom-in option”, “I think they need to record new videos, in which we can actually see the process”, “Maybe having the TA's stand a bit closer to the camera would help. Or holding items closer to the camera.”, “First person videos may be more beneficial as it would allow for closer examination of lab contents.”, “It was helpful to see which chemicals were used and how they were integrated into the lab.” from cohort 2 and “I liked being able to see the processes up close.”, “The diagrams, close camera angle, the attention to detail, and the explanation of each step were instructive.” from cohort 1 (14), support this recommendation.
4. Having subtitles, captions, important notes, and disclaimers pop up during the edits further reiterates important points to increase retention and makes the video more interesting and interactive. They also increase inclusivity as the material is delivered via both audio and visual means. This recommendation was supported by student comments like “I suggest adding subtitles off to the side. It would be really cool if, while the person was talking about what they are adding/subtracting to a test tube or whatever, there was a list compiled off to the side. This will help the user know exactly what's happening.”, “Labeling of things in the video or captions would be very helpful as well.” From cohort 2 and “I mean they were pretty detailed about what to do which was great. I liked how there was text sometimes to explain what was happening/going to happen. I figured out the subtitles worked a little more than halfway through the lab, and that helped with retention of information (at least I think it did).”, “The extra diagrams and notes that would appear in the videos from time to time”, “I do enjoy when pictures, slides, or texts were provided in the video. I like to read supplementary aid as I retain the material.”, “words and figures on the screen, the transition music was entertaining” from cohort 1.
5. Minimizing the length of the videos and maximizing the information in the VR videos may be desirable. However, a few students provided valuable arguments to further reduce the length of the videos via comments summarized in Supplementary Appendix B.5. Most students suggested having videos around 10 min through comments like “Incorporate editing into the video to make them not lengthy and more engaging. Stand-alone videos just feel like another lecture, but worse.”, “I think shortening the videos to show only the new parts of the lab rather than doing the whole entire lab would be way more helpful and engaging.”, “10 min was perfect”, “Some videos could've been shorter but overall 5–8 min is a good length.”, “The length was fine for some but I think the first one was over 10 min and that seemed a little long”, “Some of the videos were good length-wise, but 1 of them was way too long (16 min I believe). If there are videos they should be around 5–8 min max.”, and “The video length should not exceed 10 min”. Several other studies have also previously reported that shorter videos of approximately 10 min have been successful in increasing engagement, retention, and potential for future use (32–37).
6. Enhancing the interactivity in the videos may increase the students' reception towards VR. Student comments like “Find a way to make it more hands on VR, maybe program something where students can do stuff or see the video better”, “If the videos could have interactive segments so we had something like a virtual lab that would be very helpful for retaining info.”, and “An interactive VR might help more than just sitting and watching a video.” Support this notion.
7. Most students also revealed their desire to have the VR videos available as a pre-lab exercise. Cohort 1 students had access to videos that they could replay, rewind, and rewatch the videos as they preferred allowing them to work at their own pace (14). Cohort 2 students watched the videos in the lab. They spent extra time within the lab and had limited time to be able to go over the video or replay and rewind it. That not only took away lab time, increased the time spent in the lab but also gave less time to students to engage with the video.

Having the videos at the students' disposal may also result in repetition which further enhances retention. To that extent, the cohort 2 students commented that “I think it was effective as an overview of the process but I feel like it would've been better as a pre-lab video and not during lab time.”, “I think it would be more helpful to have these as normal videos that we can watch at home before the lab.”, “Upload the videos to blackboard so students can comfortably watch them either before or during lab.”, “I didn't find the VR beneficial; I think the videos might have been more helpful if they had been posted to BB so we could watch them before lab and have a better grasp of what we were supposed to be doing before we got there. But I'm not sure they were really necessary, since the TA's ended up explaining everything in the video again after we finished watching it.”, and “It lengthened the amount of time we spent in lab. Some labs should have taken around 2 h, but we took 3 while everyone finished watching the videos and actually understood what was happening.”.

8. Detailed assessment of which aspect of the lab may benefit the most from the incorporation of VR and optimization of the type of content delivered by the VR may enhance its applicability. In this study, VR videos were more helpful in going over the lab procedures while a traditional method was more effective in explaining background information and lab concepts. This notion was corroborated by the quantitative data and student comments from pre-and post-VR surveys: “I think it will be beneficial to encorperate both a traditinoal pre-lab introduction component along with the VR videos. The VR videos will most likely be more helpful in how to perform the steps of the procedure while the concepts and objectives are explained well by the TAs.”, “I think that there should be videos for the pre-lab before introducing VR into the lab so students can get a better grasp on material.”, “I think demonstrating the techniques in VR is helpful but I think explanation of the concepts and ideas out of VR would be beneficial.”, and “I think a combination of both VR and traditional methods would be good. One way to encorperate both might require the VR videos to be shorter. Maybe shows how the lab is performed in VR while providing information in traditional format”.
9. Incorporating the most helpful and desirable characteristics of the traditional methods into the VR experience may enhance its reception among students. Students revealed via the comments that the most valued attributes of traditional labs are in-person interaction with the lab instructor, being able to ask them questions, and seeking help from the instructors during the procedures. Student apprehensions as alluded to in their comments revealed their perception of reduced interaction with and help from the lab instructors after the incorporation of VR. A balance of novel technology and traditional methods to ensure the comfort of the students may smoothen the process of VR incorporation into biomedical engineering labs.

## 5 Conclusions

Overall, this study aimed to assess the student perception of incorporating VR technology as a complementary mode of teaching in biomedical engineering labs. It also assessed the utility of VR-based labs in terms of student engagement, potential for future use, understanding and retention of material and tasks, and usability. While the students proceeded with apprehension and less hope about the incorporation of VR, their self-reported experience with VR also seemed unfavorable. However, the student scores on quizzes and lab reports suggested that VR may be helpful in visual demonstration and understanding and retention of the lab procedures while the traditional teaching methods may be more suitable for explaining lab concepts. On comparing the data from the two cohorts, the advanced equipment seemed to reduce the discomfort associated with watching VR videos. The Likert-type questionnaire and further insights obtained from cohort 1 and cohort 2 open-ended responses also revealed that apart from equipment quality, content quality, and teaching style may impact the experience of students with VR. Student responses along with the Likert-type questionnaire reveal several key factors that may contribute to the more effective incorporation of VR technology as a complement to traditional learning methods. Future studies are required to further validate the utility of the aforementioned factors and recommendations via a quantitative assessment. Finally, this study helped further our understanding of the successful incorporation of VR videos in traditional biomedical engineering labs.

## Data Availability

The raw data supporting the conclusions of this article will be made available by the authors, without undue reservation.
