# Supplementary material for: Immersive virtual reality-based learning as a supplement for biomedical engineering labs: challenges faced and lessons learned
Source: Front Med Technol. 2024 Mar 19;6:1301004. doi: 10.3389/fmedt.2024.1301004 (PMC10985327; doi:10.3389/fmedt.2024.1301004)
Supplement: Supplementary file 1 [file Datasheet1.docx]

**Table A.1:** Lab report requirements and grading rubric

| **Criteria** | **Std Points** |
| --- | --- |
| **1. General Format** Title, name, date, length, and margins. | **5** |
| **2. Introduction: background information**  Background information is provided and clearly stated Objectives of the experiment are stated clearly in paragraph form. | **5** |
| **3. Materials and Methods** Specific list of materials and their amount (where applicable). Specific procedures are listed in complete sentences, passive voice, and in paragraph form. Copying the methods from the lab protocol will result in zero points. (1 mark will be deducted for each statement not made in the past tense or passive voice) | **10** |
| **4. Results** Data presented in table or figure format if necessary and stated in complete sentences in paragraph form. Tables and figures should be appropriately labeled, cited in text and completely andproperly explained. (1 mark will be deducted for each unlabeled, uncited figure) | **10** |
| **5. Discussion and Conclusion** Results are summarized and analyzed. Points to discuss in the lab report are all mentioned. Experiment objectives are addressed. Mention “why” the results were obtained in a certain. Mention what went wrong and why that happened. Cite external resources that support the obtained results or contradict them. | **10** |
| **6. Conclusions** Summarize the most important aspects of your report (objectives, methods, and results) … very briefly (in a nutshell) | **5** |
| **7. Writing Quality** The report should be well organized, cohesive, and written in clear language. Report should not contain spelling or grammatical errors. (0.5 marks will be deducted for each spelling and grammatical error) Report should state the references properly. | **5** |
| **Total** | **50** |

**Table A.2:** Questions present in the survey pre-VR

| **5-point-scale Likert question** | |
| --- | --- |
| Perception about VR | I think the Virtual Reality (VR) videos will help me feel more engaged with the lesson. |
|  | I think the VR videos will help my retention of the course material. |
|  | I believe the VR videos will be helpful in learning the course material. |
|  | I think the skills/techniques in the VR videos will be transferable to real life. |
|  | The novelty of VR videos would make course material more interesting. |
| Experience with Traditional Labs | In the previous labs, I found the traditional lab introduction helpful in understanding the purpose of the lab. |
|  | In the previous labs, I found the traditional lab introduction helpful in understanding the procedures of the lab. |
| Familiarity with VR | I feel comfortable/familiar with VR equipment. |
| **True/False questions** | |
| Previous Experience with VR | I have previous experience with Virtual Reality (VR) equipment before. |
|  | I have experienced some kind of discomfort (e.g. claustrophobia, nausea, dizziness) while using VR technology. |
| **Open Response questions** | |
| What are your expectations for the VR videos? | |
| What about the traditional pre-lab introduction is helpful or not helpful? | |
| Suggestions or Comments? | |

**Table A.3:** Questions present in the survey post-VR

| **5 point-scale Likert questions** | |
| --- | --- |
| Engagement | The use of VR helped me feel more engaged with the lesson. |
|  | The use of VR technology eliminated or reduced auditory and visual distractions from the environment. |
|  | The length of the videos was appropriate for the material covered. |
| Content | The VR videos increased my retention of the course material. |
|  | The videos provided enough information to understand the task. |
|  | The use of VR technology helped me understand the material. |
| Potential for Future Use | I felt confident applying the skills/techniques from the videos in the lab. |
|  | I would like to use this kind of video in future labs. |
|  | The use of videos met my expectations about this lab. |
| Functionality | I experienced some kind of discomfort (e.g. claustrophobia, nausea, dizziness) while using the VR technology. |
| **Open response questions** | |
| Did you experience any problems using/viewing the videos for the lab? If so, which ones? | |
| Please comment on the video length. | |
| What aspects of the VR lessons were helpful and/or effective? | |
| What aspects of the VR lessons were not helpful or effective? | |
| Suggestions or comments? | |
