# Supplementary material for: Immersive virtual reality-based learning as a supplement for biomedical engineering labs: challenges faced and lessons learned
Source: Front Med Technol. 2024 Mar 19;6:1301004. doi: 10.3389/fmedt.2024.1301004 (PMC10985327; doi:10.3389/fmedt.2024.1301004)
Supplement: Supplementary file 2 [file Datasheet2.docx]

B.1 Students revealed their low expectations from VR-based labs with phrases like “no expectations”, “Pretty low expectations and they probably will not help more than regular videos or in person instruction”, “Very low to be honest”, “I don't really have any.”, and “disappointing”. Some of the students also expressed their apprehensions concerning the time and learning curve associated with adapting to VR technology with comments like “I expect them to take some getting used to. The labs might take longer then usual as a result.”, “It will be difficult to use and the lab material will get lost in the confusion of trying to get it to work correctly.”, “I do not have many expectations other than that the novelty of VR will capture attention, however I belive that it will overcomplicate things and take longer than traditional methods.”, “I expect them to make me dizzy and that I won’t really know how to use it”, and “Most likely confusing”.

B.2 A few students weighed in on where they see the utility of VR videos as opposed to the traditional lab methods via comments like “It is only helpful when it is not rushed. Sometimes teacher can rush through instructions instead of breaking down what we are going to do.”, “I think more visuals would help us understand the goal of the lab.”, “The traditional pre-lab introduction is helpful for going over the objective and concepts of the experiment as well as the procedure. I will say that it is somewhat difficult to visualize what we are actually going to be doing if the steps are just described and not shown.”, “I think you can relate the quetions of the quizzes with the things we see or read in the videos.”, and “I like the pre-lab introduction because we really get to find out the overall objective for the lab and the TAs give a brief introduction on what we'll be doing that day in lab. This allows for us to really get our minds into what we're supposed to be doing and helps throughout the rest of the lab. With the current introduction 90% of the time it is helpful, but sometimes it is easier to see the experiments be done and then do it ourselves.”.

B.3 The students also revealed the specific features of the traditional labs they liked via comments like “It's helpful to have bullet points and a TA to ask clarifying questions”, “I think that the intro done at the beginning of class is helpful just to solidify the concepts and procedures that we will be performing”, “It is in person usually and was very helpful because we could ask questions.”, “I think being able to ask questions is a very large part about feeling comfortable doing the procedures for lab.”, “Our TA can stop and ask if we understand and either explain it differently or move on”, “Having the slides to look at about the experiment is very helpful in lab and for the report.”, and “The one-on-one conversation with TAs”.

B.4 The following comments are derived from the post-VR survey “What aspects of the VR lessons were helpful and/or effective?”, “What aspects of the VR lessons were not helpful or effective?”, and both pre- and post-VR survey question “Suggestions or comments?”.

Cohort 2 student comments included “Overall, I found the TA's explaining everything in person more helpful than the VR”, “I feel like we need better explinations on why we conduct the labs. Labs are interesting but I find it hard to write a lab report because we aren't explained why we're doing the lab”, “I think the TAs explaining the background on the videos was not helpful because I didn't know what they were talking about.”, “Be very general in the descriptions. We have the lab doc for details, just go over the tools used, what is gonna happen, and focus on the visuals instead of the technical content”, “I think a video on what we are trying to accomplish would be more beneficial than watching the TAs pipette”, “They were concise and informational. So much so that not a lot of questions needed to be asked to understand the task.”, “The video didn't show much more than people picking things up saying what they were and doing some pipetting pretty much. It was not helpful. Video is not clear enough to distinguish between what is being handled beyond what is said. Also sometimes it is hard to make out some of the more technical words.”, and “Be very general in the descriptions. We have the lab doc for details, just go over the tools used, what is gonna happen, and focus on the visuals instead of the technical content”.

In contrast, cohort 1 student comments included “I mean they were pretty detailed about what to do which was great. I liked how there was text sometimes to explain what was happening/going to happen. I figured out the subtitles worked a little more than halfway through the lab, and that helped with retention of information (at least I think it did).”, “explaining why we were adding which chemicals was helpful.”, “being very descriptive”, “The content of the videos was well thought out and helpful.”, “The explanation of the concepts in the beginning. This part was most helpful for overall knowledge and the quizzes”, and “The intro part and the part of the experiment where the TA explained the nuances of how to pipet or something hard they were doing in the video.” [9].

B.5 Students provided valuable arguments to further reduce the length of the videos via comments like “I think that some of the lab videos were very long, and I have very bad ADD/ADHD and the long videos really don't help because I cannot concentrate that long.”, “I somehow wished the length of the video could be short but I do agree that the videos covered everything needed for the lab.”, “It was harder to remember all the steps to the lab when the videos were long”, “I felt that in most cases the videos were too long. I think they could have been edited especially for steps that were repetitive.”, “The first few videos were far too long. It doesn't make sense for us to memorize a 16min video, it's too much information.”, and “Lab 8 video length was too long and made me bored and not retain the information well”.
